# Vitamin D Signaling in Inflammation and Cancer: Molecular Mechanisms and Therapeutic Implications

**DOI:** 10.3390/molecules25143219

**Published:** 2020-07-15

**Authors:** Ahmed El-Sharkawy, Ahmed Malki

**Affiliations:** 1 Human Molecular Genetics Laboratory, Institute of Genetics and Biophysics “A. Buzzati-Traverso” (IGB)-CNR, 80131 Naples, Italy; ahmed.elsharkawy@igb.cnr.it; 2 Biomolecular Science Programme, Università Degli Studi Della Campania “Luigi Vanvitelli”, Viale Abramo Lincoln, 5, 81100 Caserta, Italy; 3 Biomedical Science Department, College of Health Sciences, QU Health, Qatar University, Doha 2713, Qatar

**Keywords:** vitamin D, carcinogenesis, inflammation immunomodulation, signaling pathways, cell death, therapeutic implications

## Abstract

Vitamin D and its active metabolites are important nutrients for human skeletal health. UV irradiation of skin converts 7-dehydrocholesterol into vitamin D3, which metabolized in the liver and kidneys into its active form, 1α,25-dihydroxyvitamin D3. Apart from its classical role in calcium and phosphate regulation, scientists have shown that the vitamin D receptor is expressed in almost all tissues of the body, hence it has numerous biological effects. These includes fetal and adult homeostatic functions in development and differentiation of metabolic, epidermal, endocrine, neurological and immunological systems of the body. Moreover, the expression of vitamin D receptor in the majority of immune cells and the ability of these cells to actively metabolize 25(OH)D3 into its active form 1,25(OH)_2_D3 reinforces the important role of vitamin D signaling in maintaining a healthy immune system. In addition, several studies have showed that vitamin D has important regulatory roles of mechanisms controlling proliferation, differentiation and growth. The administration of vitamin D analogues or the active metabolite of vitamin D activates apoptotic pathways, has antiproliferative effects and inhibits angiogenesis. This review aims to provide an up-to-date overview on the effects of vitamin D and its receptor (VDR) in regulating inflammation, different cell death modalities and cancer. It also aims to investigate the possible therapeutic benefits of vitamin D and its analogues as anticancer agents.

## 1. Introduction

Vitamin D is a fat-soluble pro-hormone, represented mainly by two compounds: vitamin D3 (cholecalciferol) and vitamin D2 (ergocalciferol). Both are considered as substantial nutrients for human health. Vitamin D3 can be obtained from several dietary sources including, eggs, fish, meat and dairy products [1]. Moreover, it is made naturally in the human body following exposure to ultraviolet light.

Vitamin D roles in the regulation of calcium-phosphate homeostasis and in controlling bone turnover are very well documented. These roles start early during growth and continue into adult age. Insufficient vitamin D status results in acceleration of bone turnover, reduction in bone density, and increases the possibility of bone fractures. During growth, vitamin D deficiency leads to rickets [2] and in adult age causes osteomalacia [3]. Additionally, low vitamin D status causes bone fractures as bone turnover increases and bone density decreases. Apart from these well-known effects on skeletal and bone health, research in the past two decades has revealed that vitamin D also has a myriad of pleiotropic effects in different physiological settings. Thanks to the discovery that the expression of vitamin D activating enzyme 1-α-hydroxylase (CYP27B1) are not restricted to bone and kidney, but also expressed in other organs, including intestine, prostate, pancreas, and platelets [4]. Moreover, several immunologic cells express vitamin D receptor (VDR) and CYP27B1, providing strong evidence for the roles of vitamin D in regulating immune functions [5].

In 1863, Rudolf Virchow proposed an idea linking inflammation and cancer when he noticed that chronic irritation causes cancer [6,7]. Approximately 50 years later, his student Yamagiwa experimentally proved that chronic inflammation causes cancer [8]. Since then, dozens of studies employing molecular biology techniques and genetically-modified mice were done and revealed the importance of chemokines, cytokines, growth factors and inflammatory cells in cancer-related inflammation [6,7] These studies have laid the foundation for the pro-tumorigenic effects of inflammations and helped in unraveling the mechanisms through which inflammation causes cancer.

Recent epidemiological and clinical data strongly suggest a correlation between deficient or low serum levels of vitamin D and increasing the risk of developing multiple cancers [9]. Garland and Garland in 2006 proposed that colon cancer is linked to vitamin D deficiency when they studied the geographical distribution of deaths from colon cancer in the United States. They found that mortality rates were higher in areas receiving low amounts of natural light as in the case of major cities or high latitudes areas [10]. In 1981, Colston et al. presented the first evidence on the inhibitory effects of the active form of vitamin D on tumor cells [11]. They found vitamin D receptors in malignant melanoma in both cultured cells and melanoma tissue and that these inhibitory effects were dose-related. In the same year, Abe et al. reported the ability of the active form of vitamin D (1,25(OH)2D3 or calcitriol) to differentiate leukemia cells (HL60) into macrophage lineages [12]. Since then, many in vitro and in vivo studies were conducted and confirmed the anti-cancer properties of calcitriol [13,14,15,16,17,18,19,20,21,22,23,24,25,26,27].

These antitumor properties of active vitamin D and its analogs are principally mediated by binding of vitamin D-vitamin D receptor (VDR) to DNA and subsequent genomic regulation of target genes and related pathways. These effects include inhibition of malignant cell proliferation, induction of differentiation and apoptosis, inhibition of angiogenesis, invasion, metastasis [28] and inhibition of cancer-related inflammation [29]. Moreover, preliminary data suggest that calcitriol and other vitamin D analogues are promising in targeting and inhibiting cancer stem cells (CSC) of prostate and breast malignancies [30].

The aim of this review is to give an up-to-date overview of the antitumor properties of vitamin D and its analogs. Additionally, it aims to unravel potential underlying mechanisms giving special attention to immune-modulatory effects of vitamin D in cancer-related inflammation. The therapeutic potential of vitamin D and its analogs in the prevention and treatment of different malignancies will be also addressed.

## 2. Epidemiology

Epidemiological data confirmed a significant association between vitamin D deficiency and the high risk and poor prognosis of multiple tumors [31,32,33,34]. Since the first observation by Garland et al. [10] linking vitamin D low levels and the high incidence of colon cancer, a large number of meta-analysis were conducted and verified this hypothesis including studies on lung, prostate, breast and other cancers [35]. The results of these studies confirmed the inverse correlation between low circulating levels of vitamin D and the high risk of total cancer and that vitamin D has protective effects against cancer of multiple sites as shown in a large case-cohort study within the Japan Public Health Center-based Prospective Study [36]. A meta-analysis of 25 studies and 17,332 cancer patients showed better outcomes for those patients with higher circulating levels of vitamin D at or near the time of diagnosis with 10 nmol/L increase confers a 4% reduction in cancer specific mortality [37].

Vitamin D also has beneficial roles in preventing and treating some gastrointestinal cancers [38]. In a study assessing the correlation between oral intake of vitamin D and colorectal cancer, the authors found that individuals consume 1000 IU/day or more oral vitamin D or 33 ng/mL (82 nmol/L) or more serum 25-hydroxyvitamin D had 50% lower incidence of colorectal cancer compared to individuals with less than 100 IU daily vitamin D intake [39].

Ma et al. conducted a meta-analysis of nine studies on vitamin D intake and another nine studies on serum 25(OH)D levels and found that high-dose intake of vitamin D and high levels of serum 25(OH)D levels lower the risk of colorectal cancer by 12% and 33%, respectively [40].

A recent meta-analysis of studies assessing the correlation between vitamins intake and the risk of pancreatic cancer (PC) with dose–response analysis found that vitamin D intake can decrease the risk of PC by 25% [41]. Clinical studies also have revealed anti-inflammatory and inhibitory roles of vitamin D against liver cancer cells. Severe deficiency of 25(OH)D results in poor prognosis in hepatocellular carcinoma patients (HCC) [42]. Vitamin D has also been linked to the development and progression of other cancers [43]. Some studies suggested that low levels of 25(OH)D are associated with increased risk of prostate cancer (PCa) [44]. This was confirmed by a new dose-response meta-analysis using Seven cohort studies with 7808 participants and found that the reduction in mortality of prostate cancer patients associated with higher levels of 25(OH)D and that vitamin D represents an important protective agent in prostate cancer progression and prognosis [45]. However, another recent study reported an association between high vitamin D serum levels and increased risk for prostate cancer and a modest dose–response effect exists [46]. The biological and molecular mechanisms involved in this association are not clear and further studies are needed to unravel these links.

Vitamin D could also have a role in breast cancer survival. Experimental studies have showed that vitamin D possess anti tumorigenic effects against breast carcinomas, however, data linking patient’s vitamin D levels and the breast cancer survival are inconsistent [47]. Kim et al., performed a dose-response meta-analysis of 13 studies assessing vitamin D intake, 25(OH)D levels and risk or mortality of breast cancer. They showed a 2% reduction of breast cancer incidence with every 100 IU/day increase in vitamin D intake [48].

Another recent dose-response meta-analysis of a cohort of six studies with 5984 patients were analyzed and found that for a 10, 20 or 25 nmol/L increase in circulating 25(OH)D levels, the mortality risk of breast cancer decreased by 6%, 12%, and 14%, respectively [49].

Despite the efforts have been made to understand the relationship between vitamin D and breast cancer risk, there are many complex issues remain to be solved. Population studies confirmed that higher intake of vitamin D or it’s metabolite 1α,25(OH)_2_D_3_ reduces the incidence of initial cancer development and may slow down it’s progression. However, these metabolites uptake and their metabolism in vivo seem to depend on biology of breast cancer. Additionally, a little is known on how systemic vitamin D might interact with other known risk factors of breast cancer whether genetic, environmental or endocrine modulators. Also, the exact roles of vitamin D receptor in different cell populations within breast tumors (adipose, endothelial, fibroblast, immune or epithelial) [50], their interaction in normal and tumour tissue and in tumour microenvironment need to be clarified.

## 3. Metabolism

**Figure 1:**
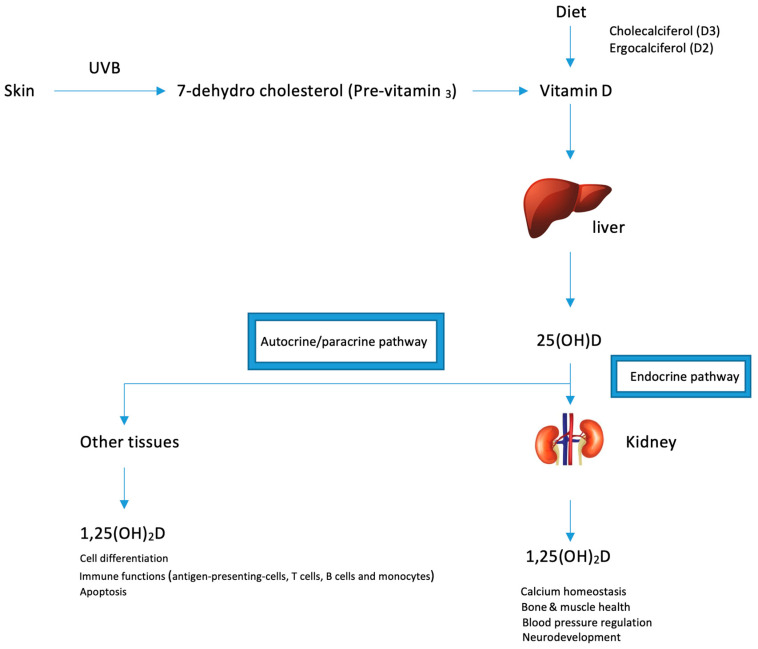
Overview of vitamin D activation and its pleotropic effects.

Vitamin D is obtained in an inactive form from sun exposure, food or supplements and in order to become activated, it must be hydroxylated through two enzymatic reactions in the liver and kidney (Figure 1). The first step of vitamin D metabolism starts in small intestine where it is absorbed with other dietary fats [51]. Once it enters the lumen, bile acids are released, which emulsify and form lipid-containing micelles facilitating their diffusion into enterocytes [52]. Once absorbed, vitamin D is packaged into chylomicrons and has to face 2 fates: a part is transported into liver, and the other fraction is taken up by skeletal muscles and adipose tissue [53]. In the liver, vitamin D binding protein (DBP)—a specific carrier protein—facilitates their transport into hepatocytes and other tissues.

Ultraviolet B radiation (UVB) converts pro-vitamin D3 (7-dehydrocholesterol) into the pre-vitamin D3 form (pre-calciferol). Pre-vitamin D3 is thermodynamically unstable and undergoes thermal isomerization into vitamin D3 in the epidermis [54]. Approximately 15% of 7-dehydrocholesterol is converted into pre-vitamin D3 upon exposure to sunlight in the skin. Further exposure to sun light will convert the pre-vitamin D3 into lumisterol and tachysterol as well as revert back to 7-dehydrocholesterol. lumisterol and tachysterol are photoproducts that have no effect on calcium metabolism. In addition, absorption of more solar UVB radiation converts vitamin D3 into several suprasterols and 5,6-*trans*-vitamin D3 and converts previtamin D3 into several toxisterols. This photodegradation mechanisms protects against vitamin D intoxication as even intense exposure to sunlight yields products that have no calcemic activity [54].

A number of factors affecting cutaneous vitamin D_3_ synthesis including the solar zenith angle, skin pigmentation intensity and ageing. Vitamin D status (total measurement of vitamin D_2_ and vitamin D_3_) is also influenced by season changes and latitude (reviewed thoroughly in 54).

The three main enzymes responsible for vitamin D metabolism are: CYP27A1, CYP27B1 and CYP24B1 which represent members of the cytochrome P450 superfamily. In the liver, CYP27A1 hydroxylates vitamin D into 25(OH)D. full activation occurs in the kidney where CYP27B1 catalyzes 25(OH)D into the active form 1,25(OH)_2_D_3_ which exerts the biological effects in humans.

Higher levels of 1,25(OH)2D3 rapidly induce CYP24A1 which in turn converts 1,25(OH)2D3 into its corresponding calcitroic acid, the water-soluble inactive form. This enzyme also found to be highly expressed in cancer tissues and promotes cancer progression as a result of vitamin D activity neutralization, which may have an anti-tumor effect [55].

Sun H et al. proposed CYP24A1 as a potential diagnostic biomarker for progression of colorectal cancer in a study of 99 patients with colorectal cancer [56]. They found that elevated protein levels of CYP24A1 induced deeper tumour invasion, lymph node metastases and venous permeation. With these observations, CYP24A1 is considered as a pro-oncogenic protein. One plausible mechanism of these effects is that increasing the expression levels of CYP24A1 results in deactivation of 1,25(OH)2D3, hence halting its antitumor effects in CRC [56,57]. Recently, Shiratsuchi et al. found that increased expression of CYP24A1 counteracts the anti-proliferative and growth inhibitory properties of 1,25(OH)2D3 in a model of lung cancer xenograft in vivo and that this catabolic enzyme could be considered as a potential oncogene in lung cancer [58]. Not just in colorectal cancer and lung cancer, CYP24A1 also has been shown to have a pro-survival stimulatory oncogenic effects in breast carcinoma cells as shown by Osani et al. [59] when they suppress the constitutive expression of CYP24A1 and found that tumor growth has been reduced significantly in vivo [59]. Luo et al. screened small molecules library to identify possible inhibitors of CYP24A1 and discovered a new protein kinase CK2 inhibitor named 4,5,6,7-tetrabromobenzimidazole (TBBz) that significantly augments 1,25(OH)_2_D_3_-mediated antitumor effects both in vitro and in vivo in prostate cancer cells and xenografts [60]. These results suggest that the inhibition of CYP24A1 both on mRNA and protein levels and finding novel inhibitors of CYP24A1 could serve as a successful strategy for increasing the anti-cancerous effects of 1,25(OH)2D3.

## 4. Vitamin D Status

Vitamin D status is the measurements of the circulating level of 25(OH)D in the blood. However, to date there is no general agreement on the threshold levels to define desirable vitamin D levels. There are a variety of techniques used to measure 25(OH)D. For example, the competitive protein binding assays and radioimmunoassays are functional in determining vitamin D sufficiency and deficiency. However, they have a lot of technical difficulties and need to be run routinely. For that reason, reference laboratories have switched to LC-MS which quantitatively measure both 25(OH)D2 and 25(OH)D3. Physicians should be aware of the total 25(OH)D, i.e., 25(OH)D2 plus 25(OH)D3 of their patient.

Guidelines from different scientific societies in different countries established 50 nmol/L or 75 nmol/L to consider vitamin D sufficiency [61,62,63]. Generally, it’s widely accepted that 25(OH)D3 levels lower than 50 nmol/L causing bone metabolism alterations, adult myopathy, and increased risk of falls [64,65,66,67,68].

According to the Institute of Medicine (IOM), the 25(OH)D blood level should be 20 ng/mL (50 nmol/L) or above, which is adequate to achieve maximum bone health [69]. In 2011 the Endocrine Society’s Practice Guidelines Committee also recommended a serum blood level of 25(OH)D of at least 30 ng/mL (75 nmol/L) to be sufficient vitamin D and required to reduce the risk of falls [62,70]. This definition has also been accepted by the National Osteoporosis Foundation, International Osteoporosis Foundation, American Association for Clinical Endocrinologists, and the American Geriatric Society [71].

## 5. Vitamin D Signaling

Vitamin D exerts its biological actions upon binding to a nuclear vitamin D receptor (nVDR) via genomic and recently identified non-genomic pathways. In the genomic pathway, when it binds to the nVDR, the vitamin D-nVDR forms a heterodimeric complex with retinoid-X-receptor (RXR) which translocates into the nucleus where it binds to the vitamin D response elements (VDRE) and triggers the transcription of downstream targets like CDKN1A, C-MYC, CDH1, and CYP24A1.

In the mid-1980s, the observation that some of the vitamin D actions were too rapid to be explained by changes at the genomic level attracted scientific interest as these non-genomic actions opened many additional questions like how these non-genomic actions are regulated, what are the receptors involved, does the nuclear receptor (nVDR) has a role and the importance of this non-genomic actions to the overall response to vitamin D in normal and pathological conditions.

The non-genomic actions of 1,25D are manifested mainly as the activation of signaling molecules, such as phosphatidylinositol-3 kinase (PI3K), phospholipase C and phospholipase A_2_(PLA_2_) and p21ras, followed by the rapid generation of secondary messengers like cyclic AMP, Ca^2+^, fatty acids and 3-phosphoinositides such as phosphatidylinositol 3,4,5 trisphosphate. This results in the activation of protein kinases, such as mitogen-activated protein (MAP) kinases, protein kinase A, protein kinase C (PKC), src and Ca^2+^-calmodulin kinase II [72,73,74]. Furthermore, the non-genomic actions include the opening of Ca^2+^ and Cl^−^ channels [75].

In cancer, the vitamin D response always regulates the expression level of VDR. Remarkably, in breast cancer and papillary thyroid carcinomas, VDR expression is found to be very high [76,77]. The expression of CYP27B1 in cancers also showed a similar trend giving a plausible explanation for the overexpression of VDR in cancer [78,79]. The high expression levels of VDR can be used as a prognostic biomarker in patients with pancreatic, lung, colorectal and prostate cancers as this high expression found to be associated with overall improvements in prognosis in these patients [80,81,82,83]. Recently, Ferrer-Mayorga et al., showed a high VDR expression in tumor stromal fibroblasts and that this high expression is associated with a better overall survival and progression-free survival in patients with CRC [84].

This stromal expression facilitates broader actions for 1,25(OH)_2_D_3_, thus improving therapeutic strategies. Moreover, Li et al. recently demonstrated a high VDR expression in human gastric tissues and cell lines compared to normal gastric tissues and that 1,25(OH)_2_D_3_ inhibits proliferation and induces cell cycle arrest in a VDR dependent manner in TMK1 cells [85].

Moreover, 1,25(OH)_2_D_3_ regulates E-cadherin in colon cancer cells with a new mechanism. 1,25(OH)_2_D_3_ induces the upregulation of cytosolic Ca^2+^ concentration ([Ca^2+^]cyt) (required for genes expression of CYP24A1 and E-cadherin) which activates Rho-ROCK-p38MAPK-MSK1 [86]. In squamous cell carcinoma cells, PI3K/Akt/ERK1/2/MAPK signaling is activated through the rapid non genomic actions of 1,25(OH)_2_D_3_ provoking apoptosis and inhibiting of the anti-apoptotic protein cIAP and XIAP [87].

Taken together, the VDR overexpression both in cancer cells and in the surrounding stromal cells emphasize the great advantage of using vitamin D and its derivatives as anti-cancer agents with a broader therapeutic window against cancer. Moreover, the non-genomic activation of VDR might synergize with the VDR-dependent genomic pathway to produce antitumor effect of 1,25(OH)_2_D_3_.

Although these previous studies have shown the important role of VDR for the actions of vitamin D, one study by Zheng et al. reported that the knockdown of VDR in prostate and breast cancer cells resulted in apoptotic cell death [88], which may indicate a controversial oncogenic role of VDR.

## 6. Inflammation

Immune modulation and inflammation are considered as important hallmarks of cancer [89,90]. The risk of development of several cancers including liver, bladder, lung, colorectal and gastric cancers is associated with chronic inflammation [91,92,93]. Progression of these tumours is regulated by the presence of inflammatory cells and other inflammatory mediators like cytokines and chemokines in the tumour microenvironment. Both local inflammation, including tumour and host-derived immune cells, infiltrating immune cells and the inflammatory protein mediators and systemic inflammation, including small inflammatory proteins and immune cells as well as cytokines interplay with tumour progression, alter the disease course drastically and influence the response to treatment [94]. Cancer-related inflammation is implicated in nearly all phases of tumour development and progression as it affects cancer cell proliferation and growth, angiogenesis, invasion and metastasis, tumour immune modulation and tumour response to treatment [94].

Vitamin D regulates the inflammatory microenvironment through several mechanisms: upregulation of MAP kinases, inhibition of NF-kB signaling pathway, regulation of the interaction between tumour cells and the immune cells to regulate cytokines levels and the regulation of prostaglandin pathway [95].

Non-steroidal anti-inflammatory drugs (NSAIDS) are class of drugs used to treat acute pain through suppression of inflammation, examples include piroxicam and aspirin. Recently, these drugs have shown to have anti-tumour effects which prove a strong relationship between inflammation and progression of tumours [96]. Many types of immune cells are expressing VDR including, macrophages, dendritic cells, B cells, CD4+ and CD8+ T cells [97]. These cells are also synthezing CYP27B1 and are able to produce the active metabolite 1, 25(OH)_2_D_3_ from 25(OH)D_3_ locally which exert its effects through intracrine, autocrine, or paracrine mechanisms [91,92,98].

### 6.1. Vitamin D and MAP Kinase Phosphatase 5

1,25(OH)_2_D_3_ promotes the expression of MKP5 which in turn, dephosphorylates and inhibits P38 MAPK activation. P38 MAPK is a stress-activated kinase and is one of the serine/threonine-directed kinases family. Once activated, it increases the production of pro-inflammatory cytokines which amplify and sustain the inflammatory response [99]. In human normal prostate epithelial cells and primary prostate adenocarcinoma cells pre-treated with 1,25(OH)_2_D_3_, UV or TNF stimulation resulted in p38 inhibition and reduced production of IL-6 [100].

### 6.2. Vitamin D and NF-κB

NF-κB is an important transcription factor regulating both innate and adaptive host immune responses [101]. It is a heterodimer composed of two subunits; p50 and p65. In the cytoplasm, it is kept in an inactive state as a hetero-oligomer through binding to its inhibitory protein termed inhibitor of NF- kB or IkB. NF-kB is crucial for the expression and regulation of genes responsible for different responses in eukaryotic cells [102]. It has been shown that 1,25(OH)_2_D_3_ is able to modulate NF-kB pathways and therefor, inhibits inflammatory responses.

In activated lymphocytes, Yu XP showed that 1,25(OH)_2_D_3_ reduced significantly p50 and c-rel following only 4–8 h of treatment. In addition, 1,25(OH)_2_D_3_ inhibits the NF-κB transcriptional activity in these cells [103]. Bao BY et al. showed that 1,25(OH)_2_D_3_ prevents translocation of p65 subunit to nucleus and subsequent binding to DNA. Since NF-kB is an important upstream regulator of IL-8, this results in decreased production of this proinflammatory cytokine which required for angiogenesis and disease progression in prostate cancer cells [104]. Schwab et al. also demonstrated that the VDR antagonist ZK 191732 decreases activity of IkBα, hence increasing basal NF-κB activity in colon cancer cells HT-29 [105]. It also prevents binding between NF-κB and DNA through suppression of p65 activation in colon cancer cells [106].

Consistent with the previous results, double knockout of VDR in mouse embryonic fibroblasts leads to a marked decrease in the basal levels of IκB compared to VDR^+/−^ as shown by Sun et al. [107]. In addition, TNF-α or IL-1β stimulated induction of IL-6 was more strong in VDR^−/−^ than in VDR^+/−^ cells giving a strong indication that cells completely lacking VDR are more susceptible to inflammation. Collectively, these results confirm that VDR has a strong role in the inhibition of NF-κB activation [107].

### 6.3. Vitamin D and Prostaglandins

Prostaglandins (PGs) are bioactive lipids involved in several biological processes ranging from normal development and tissue homeostasis to promotion of inflammation and cancer progression [108,109]. In addition, PGs induce proliferation and angiogenesis and inhibits apoptosis in a number of cancer types leading to cancer growth and metastasis [110].

1,25(OH)_2_D_3_ decreases PGs production through multiple pathways [111]. 1,25(OH)_2_D_3_ administration suppress the expression of cyclooxygenase-2 (which catalyzes PG synthesis) and increases the expression of 15-hydroxyprostaglandin dehydrogenase (which catalyzes PG degradation) resulting in overall reduction in PGs levels in prostate and breast cancer cells [112,113]. In addition, Moreno et al. showed that 1,25(OH)_2_D_3_ inhibited the expression of the PG receptors EP2 and FP mRNA levels providing additional mechanism on the inhibitory effect of 1,25(OH)_2_D_3_ on PG expression levels in prostate cancer cells [112]. Combining 1,25(OH)_2_D_3_ with NSAIDS inhibits prostate cancer cell growth at a concentration ∼2 to 10 times lower than using a single agent [113].

Moreover, 1,25(OH)_2_D_3_ inhibits the proliferation of breast cancer cells through interfering with the COX-2/PGE2 pathway. 1,25(OH)_2_D_3_ reduces the phosphorylation levels of ERK and Era and downregulates the expression of CYP1B1 [114].

Collectively, 1,25(OH)_2_D_3_ possesses strong anti-inflammatory activities in multiple cancers through different mechanisms. Since inflammation has an important role in tumour progression, using 1,25(OH)_2_D_3_ as a therapeutic agent has beneficial effects on cancer prevention and treatment.

## 7. Vitamin D signaling Within Tumour Cells

**Figure 2:**
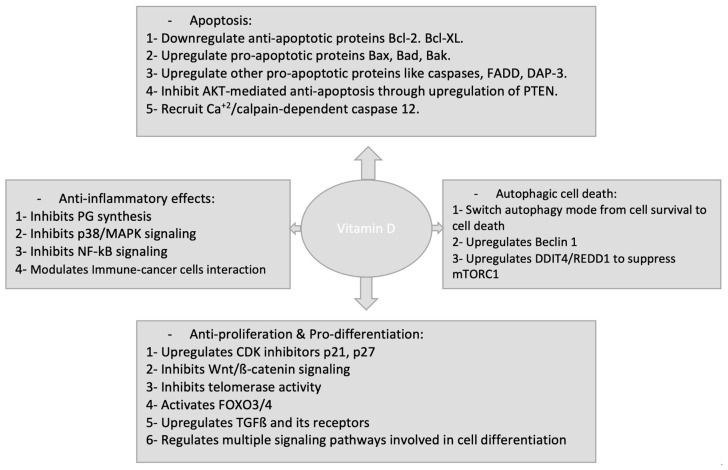
Anti-tumour effects of Vitamin D and its analogues.

1,25(OH)_2_D_3_ anticancer activities have been extensively studied in a number of cancer types. In normal cancer cells and cancer stem cells, 1,25(OH)_2_D_3_ signaling starts upon binding with VDR and exerts its anticancer activities by either regulating target gene expression or through non-genomic regulations of different signaling pathways. The actions of 1,25(OH)_2_D_3_ against cancer cells include: inhibition of cancer cell growth, induction of differentiation, induction of apoptotic and autophagic cell death and inhibition of the angiogenesis in metastatic tumours (Figure 2). Of note, recent studies have demonstrated that 1,25(OH)_2_D_3_ acts also to suppress angiogenesis, progression and metastasis in tumour microenvironment (TME) in stromal cells as well as its anti-inflammatory roles within TME.

### 7.1. Regulation of Proliferation

Several studies have confirmed the ability of 1,25(OH)_2_D_3_ to arrest cell cycle and inhibit cancer cell proliferation in many types of cancer cells; an important role for cancer prevention [115]. 1,25(OH)_2_D_3_ targets the expression of p21 and p27, both of them are critical for G1 phase cell cycle arrest and inhibition of cancer cell proliferation [116,117,118]. Wu et al. showed that the upregulation of p21 was due to sustained activation of JNK and MEK1/MEK2 pathways which act downstream the VDR through non genomic signaling [119]. P53 was also involved in regulation of P21 by 1,25(OH)_2_D_3_. Saramäki et al. showed that the promoter of P21 has multiple binding sites for P53 to cooperate with VDR for the regulation of P21 expression [116]. 1,25(OH)_2_D_3_ induced p21 high expression and induced suppression of cyclin dependent kinase 2 (cdk2) expression in TMK1 human gastric cancer cell line. These effects were dependent on the presence of mutant p53 and high VDR expression in gastric cancer tissues [120]. 1,25(OH)_2_D_3_ could also upregulate p21 even if there are no canonical VDR elements (VDRE) present in the promoter of p27 [121]. In this instance, VDR was shown to interact with sp1 to regulate the p27 promoter activity as VDR couldn’t bind p27 promoter directly [121]. Moreover, 1,25(OH)_2_D_3_ stabilizes p27 by inhibiting Skp2-mediated degradation through inhibition of Cdk2-mediated phosphorylation of p27 at Thr187 in LNCaP prostate cancer cells [122]. Li et al. also showed similar results in ovarian cancer cells [123]. Furthermore, 1,25(OH)_2_D_3_ can induce other cyclin-dependent kinase inhibitors as p15 and p16 [117,124,125].

### 7.2. Induction of Differentiation

1,25(OH)_2_D_3_- induced suppression of cancer involves also the induction of differentiation. 1,25(OH)_2_D_3_ has been tested before as a differentiating therapy in leukaemia cells. It induces differentiation of M1 and HL-60 human myeloid leukaemia cell line into mature myeloid cells [126,127]. In colon cancer cells, WNT/β-catenin signaling is activated and linked to tumor dedifferentiation and malignancy [128]. 1,25(OH)_2_D_3_ represses WNT/β-catenin signaling, therefore induces differentiation of colon cancer cells. 1,25(OH)_2_D_3_ employs several mechanisms to antagonize the Wnt/β-catenin pathway in human colon cancer. It reduces the amount of β-catenin available to bind to TCF by inducing the interaction between β-catenin and VDR [129]. Shah et al. also characterized the VDR/β-catenin in other cancer types [130].

In addition, 1,25(OH)_2_D_3_ induces high expression of E-cadherin, leading to nuclear export of β-catenin and relocation to the plasma membrane where adherens junctions present. Moreover, it induces the expression of the extracellular inhibitor of Wnt signaling, Dickkopf (DKK)-1. Collectively, 1,25(OH)_2_D_3_ functions as a multi-level suppressor of WNT/β- catenin signaling pathway [129,131,132,133,134,135].

Loss of E-cadherin induces the invasiveness of cancer cells. 1,25(OH)_2_D_3_ induces up-regulation of E-cadherin and other epithelial marker involved in differentiation such as ZO-1, as well as the inhibition of downstream targets of WNT/β-catenin signaling as c-Myc and cyclin D which results in restraining cancer cell differentiation, a feature which also involved in angiogenesis, migration and invasion.

### 7.3. Induction of Apoptosis

1,25(OH)_2_D_3_ is able to induce apoptosis in many cancer cells, including prostate, breast and squamous carcinoma cells. Induction of apoptosis in these cells often requires lengthy exposure to 1,25(OH)_2_D_3_. 1,25(OH)_2_D_3_ induces apoptosis through mitochondrial pathway where cytochrome C and BCL-2 family proteins are involved. These effects result in suppression of the anti-apoptotic proteins such as BCL-2 and BCL-XL and induction of pro-apoptotic proteins as BAX, BAK and BAD [136,137,138,139,140,141]. 1,25(OH)_2_D_3_ has been reported also to induce apoptosis in ovarian cancer cells by down-regulating the telomerase activity and decreased expression of telomerase reverse transcriptase (hTERT), effects which resulted in shortening of telomere length [142,143]. The regulatory effect of 1,25(OH)_2_D_3_ on telomerase activity was mediated by the induction of a non-coding small RNA, namely microRNA-498 (miR-498) by 1,25(OH)_2_D_3_ which decreases the expression of human telomerase reverse transcriptase mRNA [142,143].

These results revealed possible other mechanisms by which 1,25(OH)_2_D_3_ induce cancer cell apoptosis. In the same time, 1,25(OH)_2_D_3_ could also enhance the anti-tumour effects of chemotherapeutics and potentiate the cytotoxic effects of many chemotherapeutic agents as gemcitabine, paclitaxel, cisplatin and dexamethasone through different pathways [144,145,146,147]. In gastric cancer cells, Bao et al. showed that 1,25(OH)_2_D_3_ potentiates Cisplatin-mediated inhibition of cell growth and induction of cell apoptosis through decreased expression of ERK and AKT, up regulation of Bax and increased levels of P21 and P27 [148]. Moreover, Pan et al. reported that the histone deacetylase inhibitors trichostatin A and sodium butyrate and the methylation inhibitor 5-aza-2′-deoxycytidine synergistically act with 1,25(OH)_2_D_3_ to upregulate PTEN and induce apoptosis, suggesting great benefits of using 1,25(OH)_2_D_3_ in gastric cancer therapies [149]. These results strongly suggest that 1,25(OH)_2_D_3_ could be considered for combination therapy of cancer.

### 7.4. Inhibition of Angiogenesis and Metastasis

Inhibition of angiogenesis represents one of the many mechanisms used by vitamin D in its anti-cancer properties. Vascular endothelial growth factor (VEGF) is required for the formation of blood vessels which are required for growth and dissemination of solid tumours. It is involved in tumour angiogenesis and metastasis as it stimulates the survival and proliferation of endothelial cells and increasing their permeability [150]. 1,25(OH)_2_D_3_ suppresses HIF-1 both at protein and transcriptional levels and reduces the expression of VEGF in various human cancer cells including colon, prostate and breast cancers [151,152]. 1,25(OH)_2_D_3_ induces the anti-angiogenic effects through NF-κB signaling, the nuclear protein Fork headbox M1 (FOXM1) and DKK4. It was found that 1,25(OH)_2_D_3_ suppressed interleukin-8 (IL-8), an important angiogenic factor at both mRNA and protein levels. This suppression was due to the ability of 1,25(OH)_2_D_3_ to inhibit the important upstream effector of IL-8, the NF-κB signaling through blocking p65 unit from translocation to the nucleus and subsequent NF-κB-DNA binding attenuation [151]. Li et al., demonstrated that treating pancreatic ductal adenocarcinoma cells with 1,25(OH)_2_D_3_ or its analogue EB1089 (EB) downregulates FOXM1 (an oncogene that regulates cell cycle and carcinogenesis) and suppressed proliferation and metastasis of these cells [153]. Furthermore, 1,25(OH)_2_D_3_ downregulates DKK4, which is upregulated in colon cancer cells and is responsible for the malignant properties of these neoplastic cells giving a strong support on the anti-angiogenic and anti-invasiveness actions of vitamin D against cancer [133]. Finally, 1,25(OH)_2_D_3_ also regulates epithelial to mesenchymal transition and inhibits the migration and the invasion of human ovarian adenocarcinoma cell line SKOV-3. Moreover, 1,25(OH)_2_D_3_-dependent VDR elevated expression increased the expression level of epithelial marker E-Cadherin and reduced the expression of mesenchymal marker vimentin [154,155] indicating that vitamin D could be a potential therapeutic agent in ovarian cancer.

### 7.5. Induction of Autophagy

1,25(OH)_2_D_3_-dependent induction of autophagy was initially discovered in immune cells. 1,25(OH)_2_D_3_ triggers autophagy in immune cells for eradication of bacteria through the host defence peptide LL-37 [156].

An increasing body of evidences have shown that 1,25(OH)_2_D_3_ and its analogue EB1089 have the ability to suppress cancer progression through stimulation of autophagic cell death through regulating multiple signaling pathways which are critical in host defense and inflammatory responses [157,158,159] (Table 1).

Initially, Høyer-Hansen et al. reported that 1,25(OH)_2_D_3_ analogue, EB1089, caused human breast cancer cells MCF-7 to massively die by autophagy through up-regulating the tumour suppressive gene Beclin-1 [160]. The 1,25(OH)_2_D_3_-dependent induction of autophagy involves AMP-activated protein kinase (AMPK) activation triggered by activation of calcium/calmodulin-dependent protein kinase kinase 2 (CAMKK2) In these cells [161]. These effects were specifically found in luminal-like breast cancer cells and using combination with hydroxychloroquine (HCQ, an inhibitor of autolysosome acidification) resulted in augmented suppression of cancer growth in vivo [162]. Autophagy induced by 1,25(OH)_2_D_3_ or its analogue EB1089 increases also radiation sensitivity in non-small cell lung cancer (NSCLC) cells and in breast cancer cells. This augmentation of radiation sensitivity is p53 dependent as 1,25(OH)_2_D_3_ or EB1089 promotes autophagy in p53 existing breast cancer [163] and NSCLC cells [164] but not in p53-null cells, indicating the important role of p53 in vitamin D-induced autophagy. Taken together, vitamin D represents a novel magnifier of radiation responses in different human malignancies and considered as an autophagic switch in maintaining human health and in various kinds of cancer.

## 8. Therapeutic Potential and Future Perspectives

Several data from different fields including epidemiological, preclinical, clinical and in vitro experimental data strongly suggest that modulation of 1,25(OH)2D3 signaling represents a promising strategy for prevention and treatment of several kind of cancers even if confirmed clinical trials are still lacking. The dysregulated vitamin D metabolism or activity have been studied and several therapeutic interventions have been developed for cancer therapy [138]. However, some limitations restrict our use of 1,25(OH)2D3 in cancer therapy, which require better design of therapeutic strategies. One potential limitation of activating 1,25(OH)2D3 signaling systemically is the high risk of hypercalcemia, which could end in serious health effects [165,166]. To minimize this problem, research is being directed toward developing agonists of VDR which possess anticancer activities similar to those of 1,25(OH)_2_D_3_ while having reduced effects on induction of hypercalcemia [167,168,169].

### 8.1. Analogues of 1,25(OH)_2_D_3_ (Calcitriol)

Great efforts have been made to produce other analogues of calcitriol which could have greater antitumor activities and possess less potential to induce hypercalcemia, which represents the only toxic side effect of vitamin D compounds.

Each of these analogues appears to have activity in preclinical cancer models [170,171,172,173,174,175,176,177]. For example, Medioni et al., have conducted phase I and II trials of inecalcitol (TX522) with a maximum tolerated dose of 4000 μg daily. They found that this high dose is safe and maintained hypercalcemia within normal limits. Moreover, combining inecalcitol with docetaxel appears to be superior to use docetaxel alone in men with castration resistant prostate cancer [178,179].

Despite the fact these studies sound promising, neither of them have used the biologically “optimal” dose of inecalcitol, nor did the phase II trial show a sufficiently powered superior outcome to be conclusive.

Additionally, these trials failed to show clearly that an analogue has antitumor activity superior to calcitriol when using equitoxic doses of calcitriol and this analogue. Moreover, there were no preclinical studies indicate whether any of these analogues could induce less hypercalcemia than calcitriol when given at “equi-effective” doses. A plausible explanation of the apparent reduction of hypercalcemia seen in preclinical models may be due to the differences in protein binding and catabolism between the analogues and calcitriol. Demonstrating that induction of hypercalcemia requires larger doses of analogues than calcitriol does not prove that an analogue is “molecularly less hypercalcemic”.

Inecalcitol and calcitriol have different maximum tolerable doses in mice and a lower dose of inecalcitol than calcitriol is required to induce in vitro antitumor effects. However, the dose of the two compounds at which similar antitumor effects are induced, causing also similar degree of hypercalcemia [180].

Kotlarz et al. showed in a study aimed to determine the effects of adding hypocalcemic analogs of vitamin D to 5-fluorouracil (5-FU)-treated HT-29 and HCT-116 human colon cancer cells on the expression of genes related to stem-like phenotype. In HT-29/5-FU cells, the analog PRI- 2191 [(24R)-1,24-dihydroxyvitamin D_3_, tacalcitol) downregulated of genes related to survival, re-growth and invasiveness during renewal. While in HCT-116/5-FU cells, it decreased the expression of stemness- and angiogenesis-related genes. These results suggest that PRI- 2191 could be used as adjuvant therapy with conventional treatment to counteract both moderately and poorly differentiated cancer cells [181]. These results were confirmed in another study by Neska et al. using the same analogue [PRI- 2191] on colon cancer cells undergoing renewal after exposure to 5-fluorouracil (5-FU). PRI- 2191 decreased the relative expression of stemness-related genes, such as NANOG, OCT3/4, PROM1, SOX2, ALDHA1, CXCR4, in HT-29/5-FU cells. It also upregulated the expression levels of CDH1, the gene encoding E-cadherin associated with epithelial phenotype [182]. The main mechanism behind these effects is the ability of PRI-2191 to induce CDKN1A (gene encoding p21Waf1/Cip1) expression through VDR in p53-independent manner and the subsequent decrease of both the mRNA and protein level of thymidylate synthase. In addition, PRI-2191 induced E-cadherin and ZO-1 expression which contribute to the the reduction of c-Myc level and consequently the downregulation of thymidylate synthase [TS] [183].

Similarly in A549 lung cancer, the addition of PRI-2191 and sunitinib together with docetaxel showed a potent anti-tumor activities than using the compounds alone or double combinations. These effects could be attributed to the ability of PRI-2191 to inhibit tumour angiogenesis through the downregulation of VEGF-A expression and cell death induction [184]. Despite these preclinical promising results, until now no vitamin D analogue has been developed that could have the ability to induce only anticancer effects without inducing also the hypercalcemic effects.

### 8.2. Calcitriol-Mediated Anticancer Properties in Animal Models

Several studies have showed promising antitumor effects of calcitriol in in vivo models. In addition, calcitriol and its analogues potentiate the anti-cancerous effects of many other anticancer agents. These effects have been shown in several model systems of murine squamous cell carcinoma [185], as well as in several human carcinomas developed in ovary [186], bladder [187], breast [188,189], prostate [190], lung [191], pancreas [192] and neuroblastoma [193] where calcitriol or it’s analogues have essential anti-cancer properties. Calcitriol treatment in murine models of prostate and lung cancers induced significant inhibition of metastasis; which depends, in part, on calcitriol antiangiogenic effects [190,191]. Calcitriol also induces apoptosis and cell cycle arrest in tumour-endothelial cells, although these effects are not seen in normal tissues or Matrigel-derived endothelial cells [194,195]. Another effect reported by Chung et al. [196] in which calcitriol induces novel epigenetic silencing of CYP24A1 in tumor-derived endothelial cells which rendered them more sensitive to calcitriol treatment.

Inhibition of Cyp24A1 (24-hydroxylase) enhances calcitriol action, and tumours expressing high levels of this enzyme are more resistant to calcitriol-mediated actions. Calcitriol direct effects on endothelial cells may also play a role in calcitriol-mediated antitumor activity which was observed in animal models of different tumours. In vitro data are still lacking in determining which histotypes of cancer are more or less responsive to the antitumor effects mediated by calcitriol except in cases in which VDR have been lost or CYP24A1 is over-expressed [197,198,199].

#### Calcitriol Combination Regimens in Animal Studies

Mouse tumour models suggest that calcitriol acts in a synergistic way with a wide range of chemotherapeutic agents. It potentiates the anticancer properties of many cytotoxic agents includes taxanes [145], platinum analogues [146,200] and DNA-intercalating agents [201]. Optimal results are obtained when calcitriol is administered before or simultaneously with chemotherapeutic agent; however, no potentiation were observed when calcitriol is administered after the cytotoxic agent [145].

In immunodeficient mice- derived SCC and PC-3 (PCa) xenografts, pre-treatment with calcitriol or calcitriol analogues followed by paclitaxel results in enhanced antitumor effects [145,202]. In addition, in vivo studies have shown that antitumor effects of calcitriol can be also potentiated by agents inhibiting calcitriol metabolism. CYP24A1 azole antagonists enhance the antitumor actions of calcitriol both in vivo and in vitro [197,199]. An example of such agents is ketoconazole which has a significant impact on treating prostate cancer patients especially androgen-independent or castration-resistant PCa patients (have disease progression despite androgen deprivation). Ketoconazole action in prostate and non-prostate tumour cells unresponsive to androgens, indicates extra-androgenic mechanisms underlying ketoconazole activity [199,203]. Other more specific inhibitors of CYP24A1 than azoles and secosteroid have also shown high anticancer activity in in vitro and in vivo models, in addition to their action in potentiating the antitumor properties of calcitriol [204,205].

### 8.3. Vitamin D Clinical Studies

#### 8.3.1. Trials of Calcitriol as a Single Agent

Calcitriol is the biologically active form of vitamin D and the most used agent in anticancer clinical trials. It is available as an injectable (Calcijex, Abbott Pharmaceuticals, Abbott Park, IL, USA) or oral (Rocaltrol, Hoffman-Roche Laboratories Inc, Nutley, NJ, USA) formulation. As previously mentioned, calcitriol exerts its anticancer activity when used in high doses. Most limitations of using calcitriol in cancer therapy are due to its strong toxic effects in patients with cancer. However, an increasing number of clinical studies have clearly established that using an intermittent treatment schedule would allow a safe use of high doses of calcitriol.

Calcitriol oral administration on a daily schedule (1.5–2.5 μg/d, weekly dose intensity~10.5–17.5 μg/wk) is associated with a 20% to 30% frequency of hypercalcemia in men with PCa and in postmenopausal women [206,207,208,209]. However, high-dose intermittent administration schedules are used in in vivo settings.

In men with advanced PCa, daily mouth administration of calcitriol for 3 days every week (28 μg daily for 3 days) + dexamethasone (4 mg daily for 4 days) is well tolerated and considered to be safe [202]. Muindi JR et al. conducted studies using escalating doses of calcitriol (QDX3 weekly) + paclitaxel (80 mg/kg weekly for 4 weeks), as well as calcitriol (QD X3 monthly) + carboplatin (320 mg/sqm, monthly). In both studies, using 38 μg of calcitriol every day for 3 days/week and 28 μg every day for 3 days/month monthly were safely administered together with paclitaxel and carboplatin, respectively [210]. These trials showed that high dose administration of calcitriol (as Rocaltrol) was inconvenient (38 μg requires the administration of 76 caplets) and has unsuitable pharmacokinetics [211]. The high dose administration of this formulation does not lead to a proportional increase in serum levels of systemic exposure.

Beer et al. noted similar findings who used a once weekly oral regimen [212]. Fakih et al. intravenously administered calcitriol (Calcijex, Roche Pharmaceutical Corporation) with gefitinib in a weekly dose and found that very high doses of calcitriol is safe. The dose-limiting toxicity of weekly intravenous calcitriol + gefitinib was grade 3 hypercalcemia at a dose of 98 μg/wk [213]. In this regimen, phase II dose was determined to be 77 μg weekly alone and 98 μg/kg weekly when calcitriol is combined with high dose dexamethasone [213,214].

Following the administration of 98 μg calcitriol, the systemic exposure is approximately 30 ng/h/24 h. This concentration is similar to what have been reported in murine models in which calcitriol has a clear-cut antitumor activity [215].

Beer et al. have used high dose of oral calcitriol (as Rocaltrol) and found that the dose of 0.5 μg/kg weekly is very safe [212]. Also, 165 μg of DN-101 on week 1 followed by 45 μg weekly was well tolerated and produced no apparent toxicity [216]. A linear relationship between DN-101 dose and area under the curve (AUC) was found up to 165 μg.

The intermittent administration schedule (weekly or every day for 3 days weekly) resulted in hypercalcemia only at doses ~100 μg following intravenous administration. The dose of serum calcium transiently increases (11–13 mg/dL) 1 to 3 days after completion of a single or daily for 3 days schedule. However, the dose-limiting hypercalcemia has been encountered only at doses more than ~30 ng/h/mL. Hypercalciuria is universal following administration of high-dose calcitriol. Diet restriction of calcium is very hard for patients to maintain and generally it doesn’t reduce hypercalciuria. No deterioration in renal functions has been noted in patients receiving high-dose intermittent calcitriol for more than 12 months. However, there is a possibility of developing urinary tract stones occurs in 1% to 3% of patients noted by Radiographic methods (ultrasound or computed tomography) [210,211].

In PCa, trials of calcitriol and other vitamin D analogues as single agents induced only partial responses and prostate-specific antigen (PSA) responses have been seen. However, the desired anti-tumour effects were quit infrequent. Few studies using the maximum tolerated dose (MTD) of calcitriol or other analogues have been conducted. Until now, very limited antitumor activity has been seen in phase I and II trials due to the restrictions regarding the MTD, biological dose, optimal schedule and pharmaceutically available formulations of calcitriol.

#### 8.3.2. Other Calcitriol Analogues

Other analogues of calcitriol have been also studied but there is very limited information regarding their use in cancer treatment. Examples of these analogues are EB1089 (seocalcitol) [217,218,219], 1-α-vitamin D2 [220,221,222], inecalcitol (19-nor-, 14-epi-, 23-yne, 1,25-dihydroxyvitamin D3) [223] and paricalcitol (19-nor, 1-alpha, 25-dihdroxyvitamin D2, Zemplar) [224]. These studies failed to show any convincing evidence of antitumor activities that theses analogues could have.

#### 8.3.3. Calcitriol Combinational Studies

Clinical studies of 1,25(OH)2D3 plus other anti-cancer drugs such as docetaxel, estramustine, and carboplatinin patients with cancers indicated that the high dose (20IU/kg) of 1,25(OH)2D3 was safe and well-tolerated. In these clinical trials, 1,25(OH)_2_D_3_ is used other than vitamin D to achieve higher serum concentrations and hence, maximum antitumor effects [179,216,225,226,227,228,229,230,231,232,233,234,235,236,237,238,239,240,241] (Table 2).

Phase I and II clinical trials of calcitriol in combination with cytotoxic agents are facing the same challenges with regard to the interpretation of studies of calcitriol used as a single agent: the optimum biological dose and the limited data on the MTD of calcitriol.

Beer et al. [242] combined calcitriol + docetaxel for the treatment of patients with advanced PCa progressing despite castration. They used the commercially available oral form of calcitriol (Rocaltrol). In their phase II trial plan, they used docetaxel (36 mg/m^2^, weekly for 6 weeks) on day 2 + their phase II dose of calcitriol (0.5 μg/kg orally weekly) on day 1. In this trial, there was no toxicity observed and 30 out of 37 patients (81%; 95% confidence interval [CI], 68–94%). had a PSA response (>50% reduction on 2 successive measurements maintained for >28 days). Pharmacokinetics of this combination were indistinguishable from those obtained from single-agent therapy.

These results were promising and encouraged the Novacea Company (Richmond, CA, USA)] to develop another formulation of calcitriol (DN-101). Another 2 studies have been implemented: first, a large randomized, double-blind trial of docetaxel ± DN-101 (ASCENT I = AIPC Study of Calcitriol Enhancing Taxotere) with PSA response used as the end point [204]. In this study, 250 patients were enrolled and the PSA response rates were 63% (DN-101) and 52% (placebo), P = 0.07.

In the DN-101 group, the patients had a hazard ratio for death of 0.67 (P = 0.04) in a secondary multivariate analysis that included baseline haemoglobin and performance status [204]. The DN-101 median survival was estimated to be 24.5 months, compared with 16.4 months for placebo. 58% of DN-101 patients and 70% of placebo-treated patients (P = 0.07) have manifested some adverse effects which didn’t include either significant hypercalcemia or renal dysfunction. On the contrary, adding weekly DN-101 might result in a decrease of weekly docetaxel toxicity [243].

Based on these preliminary encouraging results that show increased survival in the DN-101 arm, ASCENT II was implemented: a 900-patient randomized, double-blinded, placebo-controlled phase III trial, in which survival was the end point.

The aim of ASCENT II was to define the presence of any survival difference associated with combining calcitriol with docetaxel aiming at achieving the US Food and Drug Administration (FDA) approval of this combination. Unfortunately, interpretation of ASCENT II faced two problematic issues in its design:

(1) The asymmetric design of ASCENT II (it was designed as a randomized study comparing the FDA approved docetaxel regimen (75 mg/m^2^/3 weeks) + prednisone (10 mg/day) + placebo versus docetaxel (36 mg/m^2^/week (this regimen was shown to be inferior to the weekly every 3 weeks docetaxel regimen) + prednisone (10 mg/day) + calcitriol (DN-101, 0.5 μg/kg 1 day before docetaxel) violates one of the essential tenets of a randomized trial design; that is, to eliminate all variables between standard and experimental arms, except one.
(2) Lack of data defining either the optimal or the maximal dose of oral calcitriol. Conveniently, 0.5 μg/kg weekly oral dose was used in ASCENT II. However, an intravenously dose of approximately 77 μg (>1 μg/kg in a 70-kg patient) is required to achieve the AUC (area under the curve) associated with the antitumor activities seen in mice.

The data safety monitoring committee noted that death rate in the investigational group (weekly docetaxel + calcitriol + prednisone) was greater than that found in the standard one (every 3 weeks docetaxel + placebo + prednisone). For these notes alongside the other concerns discussed above, it was not surprising that ASCENT II was halted in 2007.

In 2008, further analysis of this study found that all deaths were caused by progressive prostate cancer and no excess toxicity related to calcitriol administration was found (John Curd, MD, personal communication, 2008).

The results obtained from ASCENT II failed to define a role of using high-dose calcitriol in cancer therapy. These negative results may be related to inappropriate design and drug dose but not to the overall concept. Generally, the development of calcitriol as a cancer therapy has many unaddressed questions.

Considerable amount of data indicating the synergistic potential of calcitriol with other antitumor agents.

Several clinical trials of calcitriol combined with other agents like carboplatin, docetaxel and gefitinib have been conducted [213,243,244]. No unexpected toxicity was seen and the antitumor responses were documented [213,244,245]. However, only the trial with gefitinib used doses near the MTD while other trials used formulations that did not reach the MTD.

Although there are preclinical data that would support the study of combinations of calcitriol and several other antitumor agents including antimetabolites (methotrexate, cytosine arabinoside, gemcitabine), anthracyclines and anthracenediones, and topoisomerase inhibitors, no clinical trials of such combinations have been conducted.

#### 8.3.4. Vitamin D and Omega-3 Trial (VITAL)

VITAL is an ongoing large randomized clinical trial of 25,871 men and women in the US to study whether supplementing Vitamin D (in the form of cholecalciferol (2000 IU) with omega-3 fatty acid (Omacor^®^, 1 g) has any preventive effects against development of cancer, cardiovascular diseases or stroke in people with no prior history of these diseases [237,238,239,240,241].

The study has started in July 2010 with primary completion date estimated to be in November 2018 and final completion date in November 2020. Eligible participants were assigned by chance (like a coin toss) to one of four groups:

(1) Daily vitamin D and omega-3;
(2) Daily vitamin D and omega-3 placebo;
(3) Daily vitamin D placebo and omega-3
(4) Daily vitamin D placebo and omega-3 placebo.

Participants had an equal chance of being assigned to any of these four groups and a 3 out of 4 chance of getting at least one active agent. The primary endpoints used in VITAL study were developments of invasive cancers or cardiovascular complications such as: stroke, myocardial infarction or death due to cardiovascular problems. Secondary endpoints were set to be the development of site-specific cancers like colorectal, breast and prostate cancers, death from cancer, additional major cardiovascular events plus coronary revascularization.

The study demonstrated that: in regard to the analysis of primary endpoints during a median follow up of 5.3 years, cancer was diagnosed in 1617 participants (793 in the vitamin D group and 824 in the placebo group with hazard ratio, 0.96; 95% confidence interval [CI], 0.88 to 1.06; *P* = 0.47). A major cardiovascular event occurred in 805 participants (396 in the vitamin D group and 409 in the placebo group; hazard ratio, 0.97; 95% CI, 0.85 to 1.12; *P* = 0.69).

Concerning analysis of secondary endpoints: the hazard ratios were as follow: for death from cancer (341 deaths), 0.83 (95% CI, 0.67 to 1.02); for breast cancer, 1.02 (95% CI, 0.79 to 1.31); for prostate cancer, 0.88 (95% CI, 0.72 to 1.07); for colorectal cancer, 1.09 (95% CI, 0.73 to 1.62); for the expanded composite end point of major cardiovascular events plus coronary revascularization, 0.96 (95% CI, 0.86 to 1.08); for myocardial infarction, 0.96 (95% CI, 0.78 to 1.19); for stroke, 0.95 (95% CI, 0.76 to 1.20); and for death from cardiovascular causes, 1.11 (95% CI, 0.88 to 1.40). In the analysis of death from any cause (978 deaths), the hazard ratio was 0.99 (95% CI, 0.87 to 1.12).

Results of VITAL study are disappointed so far. Supplementing vitamin D didn’t reduce the risk of development of invasive cancer or cardiovascular disease compared to placebo. Additionally, no excess risks of hypercalcemia or related problems have been identified [240].

The main strength points of VITAL including large population sample, fixed daily dosing of vitamin D, achieved mean 25-hydroxyvitamin D levels in the targeted range and adherence to the regimen. However, there are some limitations including:

(1) Only one dose was tested in the intervention group (2000 IU/day);
(2) Approximately 40% of participants with no records of serum 25(OH)D levels at baseline;
(3) Lack of assessment of vitamin D status during the follow-up in most participants;
(4) Lack of information about sun exposure, indoor and outdoor physical activity and body-covering habits of participants;
(5) No increase in vitamin D doses according to BMI (2000 IU/day: low dose for overweight/ obese individuals)
(6) Low number of participants (12.7%) with serum 25(OH) levels <20 ng/mL at baseline
(7) The median period of follow-up was 5.3 years

## 9. Summary

Vitamin D signaling is involved in many cancers based on considerable amount of data. In established cancers, targeting vitamin signaling considered as a good option for treatment of cancer either as a single agent or in combination with other antineoplastic agents. Unfortunately, roles of vitamin D compounds in cancer treatment are still obscure and many studies have to be conducted to unravel the possible mechanisms. There are some approaches to ameliorate future clinical studies of calcitriol and to better understand whether calcitriol and other vitamin D analogs could serve as valuable anticancer agents:

(1) There is a need to define MTD, phase II dose of calcitriol as a single agent or in combination with chemotherapeutics and the definition of biologically optimal dose of these agents.
(2) Designation and conduction of randomized phase III trials with the analogue be the only variable.
(3) Defining Vitamin D response-dependent biomarkers, this could facilitate selection of an active dose to therapeutically study vitamin D and help in targeting patients with a higher likelihood of response to vitamin D compounds.

Overall, development of new effective analogues and testing of these analogues in combination therapy would be a promise for an effective, less toxic treatment of many cancer types. In addition, further clinical studies treating patients with suitable doses of vitamin D would extend our understanding of toxicity issues of this vitamin. These studies may result in finding economic and efficient agents for the treatment of multiple malignancies.

## Figures and Tables

**Table 1:** Autophagy-related pathways induced by vitamin D and vitamin D analogues.

Pathway	Experimental System Involved	Action of Vitamin D or Its Analogues
PI3KC3	Human leukemia cell line, HL60	PI3KC3 increased → induce autophagy [145].
mTOR	HL60	mTOR decreased → induce autophagy [145].
Beclin 1	HL60, MCF-7, human primary monocytes/macrophages, THP-1	Beclin 1 increased → increase autophagy [145,146,147].
Cathelicidin	THP-1	Cathelicidin increased → Beclin-1 increased → increase autophagy (through promotion of fusion between lysosome and autophagosome) [147]
Bcl-2	Human breast carcinoma cell line, MCF-7	Decrease inhibition of Bcl-2 on Beclin 1 → induce autophagy [148]. Decrease endoplasmic reticulum → Bcl-2 → induce autophagy [148].
Calcium	Human breast carcinoma (MCF-7, MCF10A)	Increase free cytosolic calcium → inhibit mTOR → induce autophagy [148]
Cyclin-dependent kinase (CDK) inhibitor, p19INK4D	Human head and neck squamous cell carcinoma (SCC25)	Decrease p19INK4D → induce autophagy [149]

**Table 2:** Representative clinical trials of vitamin D/vitamin D analogues intake for cancer prevention or treatment.

Participants	Regimen	Aim	Main Results	Status	[Ref.]
250 patients	Docetaxel (36 mg/m^2^, i.v. /week) for 4 weeks + oral DN-101 (45 μg) or placebo/day before docetaxel	Studying the safety and efficacy of the combination between DN-101 and docetaxel compared to docetaxel and placebo on AIPC	Oral DN-101 prolonged the survival of AIPC patients compared with placebo.	Completed	[216]
19 patients	Oral DN-101 (180 μg) on the 1st day and i.v. mitoxantrone (12 mg/m^2^) on the 2nd day every 21 days + prednisone (10 mg orally) daily for 12 cycles maximally	Studying the safety and efficacy of the combination between DN-101, mitoxantrone and glucocorticoids in AIPC	Despite DN-101 addition doesn’t add significant activity to mitoxantrone and prednisone, it seems to reduce the toxicity of mitoxantrone in AIPC	Completed	[225]
25 patients	Day 1: oral 1,25(OH)_2_D_3_ (0.5 mg/kg). Day 2: i.v. docetaxel (36 mg/m^2^)/ week for 3 consecutive weeks followed by 1-week with no treatment	Studying the safety and efficacy of the combination between the high-dose oral 1,25(OH)_2_D_3_ and docetaxel in patients with non-resectable, incurable pancreatic cancer	Using high-dose calcitriol with docetaxel may have activity in incurable pancreatic cancer, when compared to historical findings using single-agent docetaxel	Completed	[226]
18 patients	i.v. 1,25(OH)_2_D_3_ (74 mg) weekly and dexamethasone in patients with CRPC	Prevention and treatment of CRPC	i.v. high-dose calcitriol combined with dexamethasone was well-tolerated but failed clinically to produce effects in CRPC patients	Completed	[227]
935 patients	ASCENT: 45 μg DN-101, 36 mg/m^2^ docetaxel, and 24 mg dexamethasone weekly for 3 of every 4 weeks. Control: 5 mg prednisone twice daily with 75 mg/m^2^ docetaxel and 24 mg dexamethasone every 3 weeks.	Comparing the efficacy and safety of docetaxel + DN-101 to docetaxel + prednisone in a phase III trial	ASCENT treatment was associated with shorter survival than the control. the trial was halted due to more deaths in ASCENT arm	Stopped	[228]
23 patients	Oral calcitriol (0.5 µg/kg) in 4 divided doses over 4 h on day 1 of each treatment week, i.v. docetaxel (36 mg/m^2^) on day 2 of each treatment week and i.v. zoledronic acid (4 mg) on day 2 of the first and fifth week of each cycle. Treatment was administered weekly for 6 consecutive weeks on an 8-week cycle.	Studying safety and efficacy of combining high dose calcitriol with docetaxel and zoledronic acid in CRPC	The regimen was tolerated and PSA response was detected in half of the CRPC patients	Completed	[229]
63 patients	Daily oral supplementation of vitamin D (400, 10,000, or 40,000 IU per day)	Ki67 labeling in surgical prostate tissue, PSA assessment and vitamin D metabolite levels	Oral vitamin D_3_ lowered PSA levels, increased prostate calcitriol levels and lowered Ki67 expression levels	Completed	[230]
128,779 participants	Oral 400 IU vitamin D plus 1 g calcium per day	The association between vitamin D intake and lung cancer	In never-smoking, postmenopausal women, Vitamin D intake was associated with a lower lung cancer risk	Completed	[231]
54 patients	Daily high-dose inecalcitriol (40–8000 μg) + docetaxel	Prevention and treatment of prostate cancer	Addition of inecalcitriol to docetaxel encouraged a favored PSA response in prostate cancer	Completed	[179]
1107 patients	1,25(OH)_2_D_3_ (0.5 μg) + acetyl salicylic acid (75 mg) + calcium carbonate (1250 mg) (n = 209) or placebo (n = 218)	Adenoma recurrence after three-year treatment	Supplementing calcitriol didn’t reduce the risk of CRC recurrence	Completed	[232]
64 cases + 64 controls	Topical diclofenac 3% + calcitriol 3 μg/g	BCC progression	Adding calcitriol to diclofenac treatment inhibited BCC proliferation	Completed	[233]
104 patients with CRC	Daily vitamin D (1000 IU) alone or daily calcium (1200 mg) alone and in combination or placebo	APC/β-catenin pathway in normal colorectal mucosa	Vitamin D intake significantly suppressed APC/β-catenin pathway.	Completed	[234]
2303 healthy postmenopausal women	Treatment group: 2000 IU/d of vitamin D and 1500 mg/d of calcium Placebo group: received identical placebos	all-type cancer risk (excluding non-melanoma skin cancers)	Supplementation of Vitamin D and calcium did not result in a significantly lower risk of all-type cancer	Completed	[235]
2259 patients with colon adenoma	Daily oral vitamin D (1000 IU) alone or daily oral calcium carbonate (1200 mg) alone and in combination or placebo	colorectal adenoma recurrence	1- Vitamin D prevent CC recurrence among individuals with AA genotype in VDR rs7969585 polymorphism. 2- Vitamin D3 supplementation for the prevention of advanced colorectal adenomas may vary according to vitamin D receptor genotype.	Completed	[236]
25,871 participants	(1) Daily vitamin D3 (cholecalciferol, 2000 IU per day) + Omega-3 fatty acids (465 mg of eicosapentaenoic acid [EPA] and 375 mg of docosahexaenoic acid [DHA]) (2) Daily vitamin D3 (cholecalciferol, 2000 IU per day) + Placebo (omega-3 fatty acids) (3) Omega-3 fatty acids (465 mg of eicosapentaenoic acid [EPA] and 375 mg of docosahexaenoic acid [DHA]) + Placebo (vitamin D3) (4) Placebo (Vitamin D3) + Placebo (omega-3 fatty acids)	Risk for developing cancer and CVD	Supplementing vitamin D didn’t reduce the risk of development of invasive cancer or cardiovascular disease compared to placebo	Current	[219,220,221,222,223]

AIPC, androgen-independent prostate cancer; ASCENT, AIPC Study of Calcitriol Enhancing Taxotere; BCC, basal cell carcinoma; CRPC, castration-resistant prostate cancer; DN-101, a new high-dose oral formulation of 1,25(OH)2D3; i.v., intravenous administration; PSA, prostate specific antigen; CVD, Cardo-vascular diseases; VITAL, Vitamin D and Omega-3 Trial.
